# The Commencement of Benefits

**Published:** 1913-01-11

**Authors:** 


### OUR NATIONAL INSURANCE ACT DEPARTMENT.

### XXXVIII.—The Commencement of Benefits.

So much attention has recently been given in the public press to the formation of panels of doctors for the administration of medical benefit under the National Insurance Act as almost completely to overshadow the commencement of the payment of the sickness and maternity benefits. The commencement of these benefits is, however, a matter that cannot be overlooked, and it is important that all insured persons should clearly understand the conditions under which they will be entitled to claim the payments. The first condition precedent to the receipt of sickness benefit is that the insured person is incapable of work, and this incapacity must be caused by some specific disease or by bodily or mental disablement. It is necessary, however, that formal notice of illness should be furnished to the society, or to the Insurance Committee if the insured person is a deposit contributor, and it may be held that the date of the notice is to be taken as the date of commencement of incapacity. The right to sickness benefit commences on the fourth day after being rendered incapable of work. Sunday is not to be counted as a day in the calculation of the amount payable. Thus, taking the normal case of sickness benefit at the rate of 10s. a week, the rate of sick pay per day is to be 10s. divided by six—*i.e.*, 1s. 8d. Although Sunday is to be excluded in reckoning the amount of benefit payable, it is to be allowed to count in calculating the period as from which the payments are to commence. As an example, suppose a man is unable through illness to work on a Friday, if his incapacity continues and he is otherwise entitled, the sickness benefit will commence as from the following Monday.

As we go to press the question as to whether Sunday is to count as one of the first three days of sickness, when the sickness actually commences on that day, has not been determined by the Insurance Commissioners, but a public announcement is promised, and prominence will doubtless be given to the matter in the daily newspapers in the course of a few days.

It seems to be clear from the wording, as well as the intention of the Act, that an insured person shall have been away from work three clear days before the right to benefit commences. We have, however, heard it seriously contended that, if a workman is taken ill at any time before midnight on a day that he has been at work, that day should be counted as part of the qualifying period. If this construction were allowed, the effect would be virtually that of excluding the first two days of sickness only, and by so doing the charge on the insurance funds would be enormously increased and the validity of the actuarial estimates jeopardised.

### The Effect of Arrears.

Beside the actual incapacity for work qualifying for benefit there is the further requirement that has been commonly known as the "waiting period." In the case of sickness benefit the waiting period is a matter of twenty-six weeks dating from entry into insurance. This period will expire in the majority of cases on January 13, seeing that all insured persons who were actually in employment on July 15 last had to be insured as from that date even though they did not actually become members of approved societies until the end of October. There will, of course, be cases in which the waiting period will not expire on January 13, as, for instance, in the case of voluntary contributors or of those employed contributors who, happening to be out of work on July 15, did not enter insurance in the first week that the Act came into operation. There are also the cases to be considered of those young persons who by the attainment of the age of sixteen during the past five months have been automatically brought into insurance as from their sixteenth birthday.

The expiration of twenty-six weeks from entry is, however, not the only qualification in regard to the waiting period. Equally important, but much less generally understood, is the requirement that at least twenty-six weekly contributions shall have been paid. Thus, in order that benefit may accrue at the earliest possible moment, it is important that all contributions should have been paid. This means that since July 15 employment must have been continuous, or that at least some work must have been done in each week, otherwise there will be blank spaces on the insurance cards, for which the insured person will be responsible, both with regard to the employer's portion as well as his own.

Now it is definitely laid down in the Act that arrears of contributions are not to count during the first year after its commencement for the purpose of calculating the rate of sickness payable. This concession does not, however, extend to the requirement already alluded to that twenty-six full weekly contributions shall have been paid before any sickness benefit can be claimed. It is, therefore, important that all those whose insurance cards have not been completely stamped should, if they wish to be entitled to sickness benefit as from January 15, immediately take steps to secure from their approved societies special arrears cards on which stamps to the value of the contributions that have been missed can be affixed.

If the blank spaces are on the current (second) quarter card they can be filled without the necessity of obtaining an arrears card, but if they were on the first quarter's card the payment of the back contributions can only be made by means of an arrears card.

### Maternity Benefit.

The waiting period that must elapse before the payment of maternity benefit can be claimed is twenty-six weeks from entry into insurance, and coupled with this there is the further requirement that at least twenty-six weekly contributions must have been paid. Thus in the majority of cases the waiting period will expire on Monday next. It is necessary to qualify the foregoing statement to meet the case of voluntary contributors, for whom the waiting period is fifty-two weeks, with at least fifty-two weekly contributions actually paid.

The maternity benefit in the case of a man is a payment of 30s. on the confinement of his wife, or, in the event of a posthumous birth, of his widow. Where a married woman is herself an insured person the maternity benefit becomes payable on her confinement if her husband is not an insured person, and, in addition, she is entitled to sickness benefit while she is unable to work. When the husband and wife are both insured and entitled to benefit, the maternity allowance is to be paid on account of the husband's contributions and the sickness benefit will be paid from the wife's account. In the case of the confinement of an unmarried woman the maternity benefit will become payable, but the payment of sickness benefit for the four weeks after confinement is expressly excluded unless she is suffering from disease or disablement not connected directly or indirectly with her confinement.

In this connection it should be mentioned that the word "confinement" was left undefined in the Act, but it has since been defined in the Model Rules issued by the Insurance Commissioners as follows: "For the purposes of these rules, confinement means labour resulting in the issue of a living child or labour after twenty-eight weeks of pregnancy resulting in the issue of a child whether alive or dead."

One further point must be mentioned in regard to maternity benefit. The mother is entitled to choose whether she shall be attended by a doctor or a certified midwife, and the object of the benefit is to provide proper treatment for her. For this reason it is eminently desirable that the benefit should be paid as soon as possible after the confinement. When a doctor has been selected there will be no difficulty in making the payment at once, but when a midwife has been chosen the approved society will be unable to pursue this course. This fact has only been realised quite recently, and, curiously enough, it arises from the desire to provide the mother with proper treatment. Should it be necessary for the midwife to call in the assistance of a doctor his fee is to be recoverable as part of the maternity benefit, and thus the benefit cannot be paid by the society without incurring additional liability until all possibility of a doctor having to be called in has passed.

### Preparations for the Payment of Benefits.

It is hard to realise what an enormous business this National Insurance is. It is easy enough to talk in millions, or to make estimates in millions, but it is another matter when those millions have to be dealt with as an aggregate of individuals. We have good reason to know that the staffs of many of the approved societies have been working at exceedingly high pressure ever since the commencement of the Act. During the past few weeks the work entailed in the preparation of the returns to the Insurance Commissioners, on the strength of which the necessary funds are to be placed to the credit of the societies, has required the employment of many additional clerks, and the limit has not yet been reached. Nearly 25 per cent. of the cards received by one large society have had to be dealt with separately as irregularly stamped or otherwise inadmissible without inquiry.

Similar difficulties may arise at the outset in dealing with claims, but this is only to be expected at the inception of so huge a scheme; and there is no doubt that in the course of a few months the staffs of the societies will have become so proficient that delay in settlement will be quite exceptional.

One remark is sufficient to emphasise the colossal amount of clerical work involved. It is estimated on reliable authority that the approved societies formed in connection with the Collecting Friendly Societies and Industrial Assurance Companies have a total membership of approximately five millions. Now, at the lowest estimate the average claim per member will be about one and a half week in the course of a year. This means that *each week* these societies alone will have to deal with approximately 150,000 claims for sickness benefit, without taking maternity payments into consideration. Each case will have to be dealt with in such a way as to pass an audit by the Insurance Commissioners, and the necessary "declaring-on" notice, doctor's certificate, and other evidence relating to the claim will have to be collected and filed.

If the Insurance Act has done nothing else it has at least provided much work of a clerical nature, and it seems that there will be no need for extending the unemployment provisions to clerks for years to come.

### Higher Charges for Voluntary Contributors.

It should be pointed out for the sake of those who are entitled to become insured persons as voluntary contributors that the rate of contribution they will be required to pay will be increased if entry into insurance is not made before January 15.

As matters stand at the present time, voluntary contributors entering insurance at ages below forty-five pay the ordinary weekly contribution of 7d. in the case of men and 6d. in the case of women. Above that age the payments are in accordance with a scale drawn up by the Insurance Commissioners.

On and after January 15 the standard rates of 7d. and 6d. respectively only apply to voluntary contributors entering insurance at the age of sixteen. A table of the comparative rates for male voluntary contributors before and after January 15 was given in our issue of July 27 last.

In view of the fact that the time is so closely at hand when the higher contribution will become payable, we direct special attention to the matter and point out that at ages below forty-five the difference may be as much as 3d. a week and after that age is generally 1½d. a week.

NOTE:—Questions and Correspondence on all doubtful points are invited, and should be addressed to the Editor, The Hospital, 28 Southampton Street, Strand, W.C., and marked "Insurance."

